# Morphological Computation: Nothing but Physical Computation

**DOI:** 10.3390/e20120942

**Published:** 2018-12-07

**Authors:** Marcin Miłkowski

**Affiliations:** Institute of Philosophy and Sociology of the Polish Academy of Sciences, ul. Nowy Świat 72, 00-330 Warszawa, Poland; mmilkows@ifispan.waw.pl

**Keywords:** morphological computation, offloading, adaptivity, physical computation, computational modeling, free-energy principle

## Abstract

The purpose of this paper is to argue against the claim that morphological computation is substantially different from other kinds of physical computation. I show that some (but not all) purported cases of morphological computation do not count as specifically computational, and that those that do are solely physical computational systems. These latter cases are not, however, specific enough: all computational systems, not only morphological ones, may (and sometimes should) be studied in various ways, including their energy efficiency, cost, reliability, and durability. Second, I critically analyze the notion of “offloading” computation to the morphology of an agent or robot, by showing that, literally, computation is sometimes not offloaded but simply avoided. Third, I point out that while the morphology of any agent is indicative of the environment that it is adapted to, or informative about that environment, it does not follow that every agent has access to its morphology as the model of its environment.

## 1. Introduction

In this paper, I defend the claim that morphological computation, in spite of its growing popularity as an object of research (cf. References [1,2,3,4,5,6]), does not deserve the attention it has been given in recent years as a groundbreaking or novel kind of physical computation. In particular, by drawing partly on previous work [7], I show, in Section 2, that some (but not all) purported forms of morphological computation are not computational, and those that are compute in the mechanistic sense [8,9]. Consequently, there is nothing special about them as kinds of physical computation. Similarly, as I argue in Section 3, they do not play any unitary explanatory role that would be in any way uniquely different from other kinds of physical computation. I focus specifically on the idea that morphological computation makes offloading of computational tasks of biological organisms or robots either possible or easy. However, in some cases, the notion of *offloading* may be used, at best, metaphorically. As such, it may be somewhat misleading in the debate. This leads to the following consideration: while physical entities may bear information about the milieu in which they are found, it need not mean that this information is available for them to process. Thus, the mere presence of physical processes that lawfully covary with the processes in their surroundings is not evidence that they are engaged in morphological computation as computational models of the environment, in contrast to what Reference [10] claimed. These three considerations yield the conclusion that morphological computation as a grand category is either confusing or trivial: it is not a single specific kind of physical computational process, it does not play any single specific explanatory role, and it merely contributes to further confusion in the study of physical computation. Note that I do not claim that there is no morphological computation, or that one should not study it. I simply argue against the conceptual abuses found in the recent debate.

Before criticizing the (ab)use of the notion, clarification is in order. The term *morphological computation* was introduced to refer to computation “performed by the body that otherwise would have to be performed by the brain” ([11], p. 96). The definition suggests that morphological computation is functionally equivalent to physical computation in the *brain*. However, this equivalence may be misleading for some robots and biological organisms. Some (or even most) robots contain standard electronic components. Such components are neither brains nor contain any brains, so such robots cannot perform any computation in their brains, and hence, could never be engaged in morphological computation, according to this definition.

However, there is little reason to suppose that the computational processes of silicon chips in computers within such robots could not be performed by other physical parts of their bodies, for example by mechanical gears as opposed to electronic parts (exactly as Reference [2] studied). Thus, one could refine the above definition by substituting the term *brain* with the expression *electronic parts.* Nonetheless, then the term *non-electronic computation* would be sufficient to refer to physical computation performed by machinery built of non-electronic parts. After all, fully functional computers are also made of electric parts [12], as well as mechanical relays, gears, and pulleys.

It is, unfortunately, doubtful that this would constitute a refinement, because there is nothing specific about electric signals *as opposed to* gears and pulleys that could make a theoretical difference to the range of computational functions. Why would an arbitrary physical feature of computational machinery be sufficient to draw a distinction between kinds of computational mechanisms?

Granted, the physical makeup of any machinery may influence its breakdown patterns, levels of noise, useful life, speed, energy consumption, footprint, etc. These features may be indeed crucial in explaining a number of features of physical computers [13]. However, they do not license a motivated distinction between kinds of computation in any straightforward way. For example, even though vacuum-tube computers, relay-based computers, and silicon-chip computers all operate on electric signals, relay-based computers are much more prone to mechanical wear and tear than transistors in an integrated circuit. These differences, although obviously real and physical, do not seem to carve nature at its joints in order to clearly delineate the class of morphological computation from the non-morphological one.

Thus, it is not clear what could replace the term *brain* in this definition in order to make it applicable to all possible cases. Unfortunately, not all biological agents have brains, even if they rely on their nervous systems. Finding a non-trivial property shared by all nervous systems and all kinds of electronic parts is unlikely. The definition, therefore, seems hopelessly narrow. Thus, even though this original definition is still currently in use (cf. Reference [5]) in the present paper, it will be supplanted by the analysis proposed in Reference [7]. (Another interesting attempt at defining the notion formally in terms of programmable dynamical systems is found in Reference [14]. However, the formal definition offered there covers any kind of deterministic physical computation. The specific difference for so-called non-portable morphological computation is that “part of the information determining the computation is given by the physical structure of the device performing the computation.” (p. 18). This is understood to say that parameters that govern the evolution of a physical system “should not contain all the information necessary to perform a desired computational task on a Turing machine”. Nonetheless, such parameters may be even single-bit inputs to a physical finite state machine M and switch a highly complex mode of its operation. A single bit of course would not be sufficient to simulate this complex mode of operation without knowing the transitions of M. However, if you know the transitions of any discrete deterministic physical system, you have the information to simulate it on a Turing machine. It is difficult to say why M would then be unportable. Thus, the analysis offered in Reference [14] requires further refinement.)

Müller and Hoffman distinguished three kinds of morphological computation: (1) morphology facilitating control, (2) morphology facilitating perception, and (3) morphological computation proper [7]. In the first case, the physical structure of the system contributes to its motor control. An example of this is the morphological control in a passive-dynamic walker [15]. This contraption walks naturally without any dedicated computational circuits. Müller and Hoffman argue that there is little reason to consider the passive-dynamic walker as computational. The two remaining categories, in their opinion, are less controversial; indeed, one can find real examples of the physical shape of sensory receptors facilitating perception, although they still consider this case insufficiently computational. The reason given for this is quite obscure:

The problem that needs to be solved by the agent is not solely an abstract one, but lies at the interface of the physical world, which needs to be sensed, and the abstract world, where the agent needs to estimate the distances to objects.

([7], p. 15)

However, the requirement that problems to be solved computationally should be abstract themselves seems ill-motivated (in the next section, I will also briefly argue why the account of computation as defended in Reference [16] is deeply flawed, which further undermines the analysis offered in Reference [7]). Physical machinery used to solve problems is always physical and concrete, so one cannot say that a problem is concrete just because it is solved in the physical world for use in the physical world. Although people sometimes talk as if there were a natural divide between “abstract” and “concrete” problems; such talk simply indicates that they consider these problems of less practical use and greater difficulty in understanding. The divide has nothing to do with a purported character of problems per se but rather a lot to do with a subjective evaluation of its utility. Alan Turing built machinery for decrypting Enigma; was this an abstract or a concrete problem? Of course, it was both: defeating Hitler is a practical purpose, solved by studying mathematical considerations in cryptanalysis.

All in all, Müller and Hoffman seem to embrace an intuitive argument that some authors use: computers have to be somehow *flexible* or *programmable* to count as computers. Programmability is also stipulated in the informal definition of morphological computation proposed in 2007 in Venice at the first International Conference on Morphological Computing in Venice as well as well, cf. Reference [14]. This definition is as follows: morphological computation is “any process that (a) serves for a computational purpose, (b) has clearly assignable input and output states and (c) is programmable, where ‘programmable’ is understood in the broad sense that a programmer can vary the behavior of the system by varying a set of parameters”. As Reference [14], p. 10 shows, this definition implies that all physical computation is morphological. Therefore, the definition is too broad and will not be adopted here. 

The term *programmability* is vague and could be made precise in several ways. Here, the analysis offered by Reference [17] is endorsed, i.e., program execution literally involves loading instructions to a processor. However, one may understand the term in a broader fashion. For example, in Reference [14] it is understood in terms of the influence of parameters on the evolution of a dynamical system. Quite clearly, finite state machines with inputs that influence their state transitions count as programmable in such a case; in contrast, they do not count as such according to Reference [17]. 

However, an insistence on programmability makes a number of intuitively-computational devices non-computational, including ENIAC and classical artificial neural networks. For the current author, this is a powerful *reductio ad absurdum*. ENIAC was not programmable because it had to be manually rewired to perform any computation. Classical artificial neural networks are not programmed in any straightforward sense: they are trained, which changes the connection weights between the nodes. Nonetheless, it is obvious that ENIAC was a computer, so programmability cannot be the essential feature of computation. Thus, in the rest of this paper, it will be assumed that this kind of morphological computation is computational.

There are also possible hybrid architectures that use various physical media to jointly compute mathematical functions, and this constitutes morphological computation proper according to Reference [7]. However, as cases of physical computation, they are insufficiently divergent from cases of non-morphological computation to merit the theoretical attention they have been given. In the following section, a more detailed account of physical computation is offered to back up this assessment.

## 2. The Place of Morphological Computation in Nature

In this section, it is shown that morphological computation in the senses analyzed in Reference [6] is either non-computational or insufficiently divergent from other kinds of physical computation to deserve a separate term. To argue for these claims, the mechanistic account of physical computation [8,9] will be used. However, the same claim, as will also be indicated, can be defended from the perspective of the semantic view of computation [18,19,20,21]. Before presenting the mechanistic account, the modeling view on computation [16] embraced by Reference [7] in their critique of morphological computation will be shown to be critically deficient, which forces me to reject it.

Arguably one of the most influential objections against computationalism is the attempt to show that the notion of physical can be trivialized [22], cf. Reference [23]. The triviality argument was defended independently by John Searle [24] and Hilary Putnam [25], who presented a formal proof that any open physical system could implement *any* inputless finite state machine. The idea of Putnam’s proof is straightforward (for a detailed analysis, see Reference [26]). Assume that the finite state machine **F** has *n* states it can be in (which follows from the definition of the finite state machine). Assume that there is a way to carve a physical system **S** into *n* states such that these states are also ordered in the same sequence as in **F** (Putnam uses relations to an external clock for this purpose). Now, it can be shown that **F** and **S** are isomorphic, and this is all that the physical implementation of computation requires, according to Putnam (in the subsequent debate, such a view was dubbed *simple mapping account of computation*, see Reference [27]). In particular, Putnam argued that given a clock external to **S**, one can always carve **S** into a sufficient number of states, and if there are still insufficient states, they can be defined using the disjunction operator (so that a predicate *C_n_* would be defined as *C_i_* ∨ *C_j_* and refer to a union of sets denoted by *C_i_* ∨ *C_j_*). In a nutshell, this argument relies on the idea that isomorphism is insufficient to pick the appropriate physical structure (cf. Reference [28]).

If the triviality arguments are cogent, then any physical system implements any computation whatsoever because the same construction can be performed for any finite state machine, or any model of finite computation (note that this is not to be confused with the claim that any physical system implements some computation). This is detrimental to computationalism, for it would imply that ascribing computation to a physical system does not yield any significant predictions. For this reason, in the subsequent debate, various improvements over the simple view were offered, from requiring that states of physical systems should also be carved in such a way as to support counterfactual claims about other runs of programs or machines [29] or to track causal relations [30], to stressing that the Kolmogorov complexity of the mapping procedure should not exceed that of the computation posited [26,31,32]. Finally, more complex accounts were devised in order to avoid triviality, such as the mechanistic and the semantic views presented later in this section.

**Figure 1:**
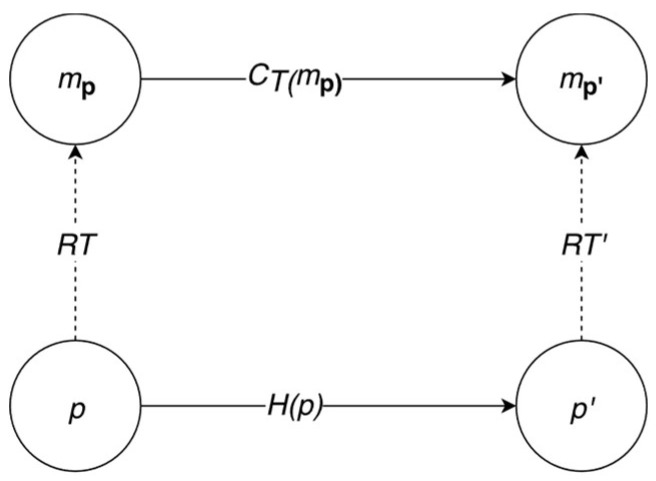
Physical computation in system **p** as modeled by relations such as R*T*.

What is, however, striking is that the account presented in Reference [16] and endorsed in Reference [7] is very much prone to triviality arguments. According to Horsman et al., physical computation is modeled by an abstract description, which should predict the evolution of the physical system, and the input values of the computation are encoded in the physical system, while output values must be decoded. However, if one follows the method presented by Putnam, then any physical system will be trivially predicted by the finite state machine because we carved the evolution of the system into so many states that they correspond to states of the finite state machine. Let me elucidate. A physical system **p** should be given an abstract representation *m***_p_** by the modeling representation relation *R_T_*. Then *m***_p_** evolves according to theory *T*, *C_T_*, resulting in the abstract system *m***_p’_**, and **p** undergoes its own evolution **H**. (In Reference [16], it is also shown that one can talk of a reversed modeling relation and define an instantiation relation, but for my argument, this is not required). This can be used to produce a diagram showing that the abstract representation corresponds to the physical system (see Figure 1).

**Figure 2:**
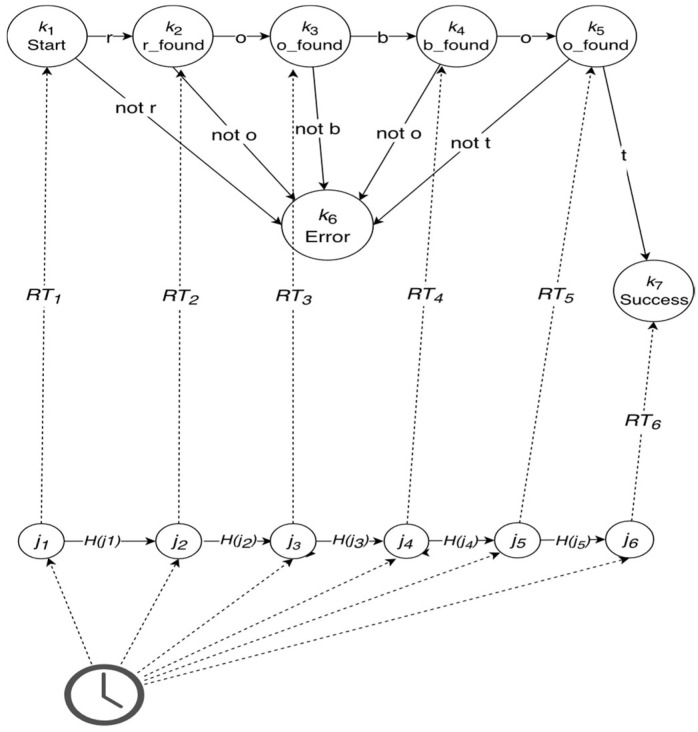
A simple acceptor finite state machine that accepts a string “robot,” mapped by six relations with physical states of **p** (*j*_1_, …, *j*_6_) that are determined by the external clock. Here, we assume that the input is “robot,” encoded by yet another clock state. Decoding of the output relies on the relationship with the clock as well.

However, there is no requirement that *C_T_* is not produced in a way devised by Putnam. Simply, if one is free to pick any physical state of **p** by using any description of **p**, there is a way to perform a Putnamesque trivialization, even for machines with input. Suppose, for example, that we wish to show that **p** implements a finite state machine F1 with a sequence of six states, *k*_1_, …, *k*_6_. F1 is an acceptor machine that accepts a string “robot.” Thus, by relying on the external clock, we can pick even the same state of **p**, *j*_1_, …, *j*_6_ at six distinct time intervals and decree that modeling relations *RT_1_*, …, *RT_6_* at these intervals obtain between *k*_1_, …, *k*_6_ and *j*_1_, …, *j*_6_. This suffices to show that **p** implements F1 (see Figure 2). Notice that the account presented by Horsman et al. does not require the input to be mapped separately. We just stipulate that the input is encoded by a relation with yet another external clock tick.

Such a procedure may be applied to any finite state machine, even without relying on external clocks (any logical relation of a physical system with a sufficiently dense set is sufficient to perform the construction). Given that the same idea may be applied to any finitely describable computational machine model, it shows that this account of computation is hopeless against this attack. While any defender of computational modeling should view Putnamesque constructions as artificial and absurd, Horsman et al. give little reason to reject them. This is because their modeling view does not really go beyond the simple mapping account, like many other attempts to formalize the notion of physical computation (e.g., Reference [14]).

The two dominant accounts of physical computation that go beyond the simple mapping account are the semantic and the mechanistic views. The first states that semantic properties are somehow essential to the nature of computation [20], and the latter views computation as specifiable without any recourse to semantic, or representational properties. In particular, the mechanistic account [8,9] considers physical computation to be a causal process that occurs in physical mechanisms, whose function is to interact with information vehicles in a way described by some mathematical model of computation (philosophers usually call such models *rules*, but models of computation need not be literally rule-based). Importantly, the information vehicles (called digits in Reference [8]) are not specified semantically; they are just bearers of structural information (in the sense of Reference [33]). They are degrees of freedom of the physical vehicle to which the mechanism is causally sensitive. Consequently, because computational mechanisms operate on vehicles whose states are specified in terms of their degrees of freedom, they are, to some extent, substrate-neutral. For the computational process to succeed, it is not essential how, exactly, the degree of freedom (say, particular level of voltage or a hole in a punch card) is physically realized, as long as the device causally responds to the change of state appropriately. In this regard, the morphology of an agent may be a vehicle of some computation. For example, one could build a Turing-complete machine from a large number of morphological NAND (NOT-AND) gates (which could be reasonably idealized as a universal machine if it were large enough for practical purposes). In other words, the mechanistic account does not exclude the existence of morphological computation by fiat. Note, also, that the causal requirement undermines the arbitrary construction such as the one in Figure 2: because *j*_1_ is not a cause of *j*_2_ and so on, the whole construction collapses.

Thus defined, computational mechanisms could form a huge class unless the notion of function is made explicit. While there are various attempts at doing so (cf. Reference [8,9,34]), the basic point is that planets, for example, do not compute their orbits. However, a person might, or an electronic computer might (if it were designed to do so). Thus, denying a physical system *S* the function to compute, leads to qualifying *S* as non-computational, even though one could find some physical vehicles as parts of *S*. Note that three mechanistic approaches to function differ considerably (see also Reference [35] for a generic account of functional mechanisms). According to Reference [8], only mechanisms whose parts were selected as types (and not particulars) by some process in accordance with a blueprint of their designs, have function (cf. Reference [36]). According to Reference [8], only mechanisms that stably contribute to goals of organisms may be computational. Both, as should be immediately clear, exclude planetary systems as possible bearers of computational functions. Finally, according to Reference [34], the function of a given mechanism is specified by a theorist in a specific explanatory context (cf. Reference [37]). While the last proposal is the most liberal, some ascriptions of function may turn out to be spurious if they lead to incorrect causal explanations [38]. However, when an arbitrary correct causal model of a mechanism is considered computational, it is difficult to discard it solely because it lacks function in the teleological sense (as References [8,9] require).

Functional considerations are, however, only a portion of the further requirements of the mechanistic account. Some defenders of the mechanistic view also require that the output of a computational mechanism be also *usable* for the finite observer by “exploiting procedures that are executable, automatic, uniform, and reliable” ([39], p. 736) (cf. Reference [8], p. 250). This requirement is, in one form or another, also common in other accounts of computation. For example, it is because of this requirement, as stated in Reference [16], that Müller and Hoffman qualify passive-dynamic walkers as non-computational [7]. According to Reference [16] (p. 15), for a physical system to be a genuine computer, not just a potential one, it is required that information is encoded and decoded from it. Thus, even if the passive-dynamic mechanical control could be re-described in terms of information processing, there would be no user of the information beyond immediate motor control. Robots such as these lack an output value that a “finite observer” could read at the output of computation. Simply, this argument presupposes that computers “process information in ways that are *useful* to us” ([40], p. 2). Note that the semantic account of physical computation is also committed to this kind of view: it requires that the inputs and outputs of a computation be semantically interpretable.

However, all this seems too quick. The assumption that the output value of computation should be available for the finite observer to read is not universally shared by all theorists of physical computation. Some of these may claim that all physical systems are, in some sense, computational [41]. This would presuppose, of course, a form of pancomputationalism, as this view is now called. Pancomputationalism is a controversial view [42,43], thus, assuming that it should be avoided by requiring that a finite observer should be able to read the output is not a critical issue.

Another, much more critical problem is that a self-regulating system might operate autonomously without finite observers and remain computational in its operation. A dramatic example is an active homing nuclear missile that could kill all biological observers by relying on its internal computational structure. If the missile is just one among many similar computational devices, operated by similar software and computer hardware, denying that it is computational seems premature. Would it cease to be computational at the moment it kills all finite biological observers? This is even more preposterous.

However, the condition that a finite observer is supposed to exploit the output of a computation plays a crucial role. One may doubt that a physical mechanism that could perform an operation, which can also be achieved through computational means, actually *is* a computational device: a morphological computer, to be exact. For example, one could use a shovel to remove obstacles that prevent rocks from falling down a hill. The same obstacles could be removed by heavy rain. Nonetheless, it would be really counterintuitive to say that the shovel, rain, or rocks (by themselves or in conjunction with a shovel or rain) are computational. 

One could appeal to functional considerations cited above to deny that rocks are computational. Even if the causal processes in this case could be re-described in computational terms, neither the rocks nor the rain has the function of computing anything. However, suppose one claims that Reference [9] or Reference [8] offer a faulty account of function, and Reference [34] seems overly liberal. What then?

Fortunately, there is another reason to doubt that rocks compute. The purported computational description of rocks is explanatorily and predictively idle [9]. This is because their basic physical description could be an equally predictive model of their behavior, thus there is no need to appeal to computation. The computational description should account for the real pattern in information processing [9,31]. Crucially, the whole phenomenon for which the hypothesized computational mechanism is supposedly responsible should be causally explainable by recourse to information processing, or to causal transaction of substrate-neutral vehicles. While this explanatory norm would require considerable unpacking to replace the usability criterion proposed by Piccinini [39], suffice it to say that passive-dynamic walker behavior depends crucially on physical interactions that are highly substrate-dependent. Simply, without the gravity of Earth, it would cease to work properly, and one could not physically replace any of its parts to process the digits (i.e., vehicles of structural information) that one could try to associate with gravity. Compare this with a simple electronic thermostat that controls a boiler: as soon as the signal exceeds a threshold, a signal is sent to open a valve. The threshold value, however, may be encoded in a variety of physical ways ranging from voltage levels to light waves in a fiber-optic cable, as long as there is an appropriate receiver of the signal that causally responds to the change in state of the physical medium. Nothing like that seems possible for the passive-dynamic walker.

Thus, the criticism offered by Müller and Hoffman turns out to be correct, albeit for different reasons than those offered in their paper [7]. Morphology facilitating control is not a case of morphological computation, or any kind of computation for that matter. However, as I show in Section 4, confusing cases of morphology facilitating control with computation proper may lie at the core of claims about the importance of morphological computation, as they have become part of the grand unifying theory of life [44]. This point is therefore important.

Unfortunately, the argument that speaks against considering morphology facilitating control computational also undermines the apparently special status of morphology facilitating perception and morphological computation proper. While morphology facilitating perception satisfies the usability condition, it also satisfies the requirement of an explanatory value of the organism’s morphological features in its functioning. Take a female cricket’s ears [45]. Their physical structure facilitates recognition of mating sounds of male crickets, so copying their morphology in a robot is a reasonable strategy for a cricket-simulating robot [46,47]. Of course, they can be built of multiple physical materials, because only structural-informational properties count in this case: i.e., the auditory information that is recognized by these highly directional ears, and the detail that is not picked up. This facilitates further processing, which does not need to not rely on extensive sound filtering. However, this is not a special class of physical computation. Attaching physical sensors to computers is not sufficient to change the physical implementation of these computers. Thus, in terms of the way computation is implemented, there is nothing special here (for an extensive analysis of the cricket robot case from the mechanistic point of view, see Reference [9]).

Morphological computation proper, as exemplified by hybrid computational systems, such as reservoir computing, is also considered computational in Reference [7]. Reservoir computing consists of a “non-linear recurrent dynamical system coupled to a single input layer and a single output layer” [48] (p. 1). Of course, one cannot deny that a system is not a computational mechanism just because it uses various physical media. Here, there is little doubt that they are computational because these systems produce usable outputs, are designed to compute certain functions (so they are functional mechanisms), and they remain sensitive to states of structural information. Nowakowski [4] argues that these hybrid architectures are paradigmatic cases of morphological computation. In his view, such architectures involve at least two parts: one capable of performing computational operations by itself, and another usable only in connection with the proper computational part (think of an electronic computer connected to some non-standard physical medium). Nonetheless, the existence of such hybrid architectures is not problematic for standard computationalism any more than the existence of vacuum tubes or various kinds of mechanical abaci. However, even if some consider the prospects of building such systems exciting and groundbreaking (as Reference [4] does), these systems do not undermine our understanding of physical computation. Hence, there is nothing special or really novel about them as kinds of physical computation. Computationally, they may all be equivalent, and because the notion of morphology remains vague, one could even claim that because all physical media have some form, morphology always plays some role in computation. (This, of course, is a slippery slope argument, but it is difficult to reject it outright as long as the role of morphology is not clearly elucidated.)

Let me summarize. In this section, it was argued that morphology facilitating control is not a case of morphological computation; that it is not necessarily computational is what both the mechanistic and semantic views on physical computation imply. The other two kinds of morphological computation analyzed in Reference [7] were shown to be credible instances of computational mechanisms, although the conception of computation in their analysis is faulty and admits triviality arguments. However, the existence of morphological computation does not seem to undermine any theoretically significant convictions. It is decidedly not novel to join analog processors with digital computers; it is standard engineering practice to design physical sensors for connection to computational mechanisms. Thus, enthusiasm about morphological computation as a novel or significant phenomenon in nature seems unwarranted.

However, it remains to be seen whether morphological computation might play a unique explanatory role, in particular in the context of embodied cognition. Perhaps it could excel there?

## 3. Explanatory Role of Morphological Computation

In this section, the focus is on the argument that morphological computation is particularly explanatorily important because it allows agents or systems to “offload” some of their computational burden to their morphology (see, e.g., papers collected in Reference [6]). The very notion has already come under some scrutiny [49]. Hybrid morphological computation is used to study the trade-offs between traditional computation and the uses of bodily structures to perform equivalent operations. By showing that morphological structures obviate the need for extensive computational processing, one could argue that the role of the body in cognition is irreplaceable. While in principle, one could try to compensate for the missing morphology by adding more computational power, such compensations are not biologically plausible (for a similar argument, see Reference [50]).

The analysis offered in this section is directed against the argument that thanks to morphological computation, computational systems can ease their computational burden by engaging their morphological dynamics instead. Because this formulation may be a bit unclear, it is important to stress that it is not aimed at analyses of various kinds of neural systems that may be more or less centralized and rely more or less on their periphery (see Reference [51]). Therefore, there could be tradeoffs in computation: some parts of the overall computational mechanism may be located in the periphery, and some in the central processing part [4]. What is under scrutiny here is another kind of argument to the effect that one can offload the computational task to the body and gain, in essence, a free computational resource (cf. Reference [52], p. 29).

Let me, therefore, analyze this argument (referred to later as MC-FREE) in detail:

1. Morphological computation may be used to direct the computational load outside the main processing unit.
2. If the computational load is moved outside the main processing unit, it offers a free computational resource.

____________________________________________________________________________

Conclusion: thus, morphological computation offers a free computational resource.

Of course, the argument’s conclusion is, to some extent, metaphorical; no resource is completely free. For example, there are energy costs related to any informational transfer and one kind of physical media may be more physically reliable or durable than another. However, what is at stake is the claim that morphological computation is particularly important for efficiency of biological computation. This would surely make it a special kind of physical computation, and it would also vindicate some of the claims of embodied cognition.

Here is a simple counterargument: Paul built a simple robot, whose physical morphology realized an XOR-gate [2]. It is highly doubtful that the robot is much more computationally efficient than one built using only electronic parts. It immediately transpires that the use of mechanical parts cannot really lead to faster computation. After all, the major breakthrough in computational technology was the use of electronics [12]. Thus, the argument given above must be incorrect. I do not deny premise (1), but (2) is immediately in conflict with the XOR-gate robot: the resource is not really free, as it requires more energy and time to work. Moreover, morphological computation is not general purpose in this case. In general, it could be argued that morphological computation cannot be easily adapted to another environmental niche, which means that it comes with yet another cost: the lack of adaptability, compared to the main processing unit computation. (I owe this observation to an anonymous reviewer of this paper. Note that this additional cost of computation pertains to other uses of physical systems for computation. For example, Lloyd notices that for quantum computers simulating a physical system directly, “the efficiency of a simulation depends on how hard it is to set up the simulator-system correspondence, to control the simulator to perform the simulation, and to extract its results” [53], p. 1074.)

In general, MC-FREE is invalid for an obvious reason. Standard computational complexity theory, which assesses the most pessimistic time complexity of a given algorithm, in terms of the number of steps required to execute the algorithm in order to process a certain number of inputs, can safely abstract away from hardware speed. This is because time complexity depends only *linearly* on the hardware speed (usually given as a constant of linear speedup in complexity analyses), and multiplicative and additive constants are negligible in computational complexity theory [54]. Thus, strictly speaking, the change of hardware, from the point of view of complexity theory, is a negligible factor. The same goes for the memory requirements (so-called space complexity).

Of course, in practical applications, differences among various kinds of physical hardware may play an important role. For example, the IBM 7090, a transistorized version of the IBM 709, featured in Stanley Kubrick’s *Dr. Strangelove*, ran six times faster and was 50% smaller, consuming 70% less power. Clearly, if their costs were equal, buying the slower and more energy-costly version would be irrational. Moreover, it cannot be denied that there may be changes in physical hardware that would not only influence the speed of operation or energy consumed, but also the range of functions computed. By replacing the IBM 709 with any IBM supercomputer produced in 2018, Dr. Strangelove would vastly increase the range of functions he could compute to inflict nuclear disaster on humanity. Still, he still could not solve really computationally difficult problems, whose complexity becomes intractable even for small sets of inputs.

One could still respond to my line of argument in the following way: standard complexity theory is not obviously applicable to biological cognition because evolution may ignore the most pessimistic outcomes if they are relatively unlikely. Moreover, biological agents usually deal with small sets of inputs, which requires a more nuanced analysis of computational complexity [55]. While this objection has some truth, it not only misses the point, but fails to prove that another kind of physical machine becomes a free computational resource; neither does it undermine my claim that the change of the underlying physical hardware can, at most, linearly affect computation speed or memory requirements.

Yet some defenders of morphological computation may appeal to results known from other fields of non-conventional computing. The idea of using physics directly to compute lies at the core of Feynman’s notion of quantum computing [56]. It was argued that a universal quantum computer cannot be fully simulated on a universal Turing machine (see References [53,57]) and it is well-known that some quantum algorithms outperform classical algorithms (cf. Reference [58]). A similar claim has been made in Reference [14] with reference to a system that simulates interaction between chemical particles:

In general, the computational cost of simulating the interaction among *n* particles is *O(n*^2^*)* in CPU [central processing unit] time and *O(n)* in memory. In morphological computation, by contrast, the inherent parallelism of the interactions means that the time necessary for computation is basically independent of the number of particles. Time is only needed for the preparation of the input; this time usually scales linearly with *n*.

([14], p. 20)

Thus, the direct use of the physical system may dramatically influence the efficiency. While this undermines the classical computational complexity argument that hardware matters only negligibly, even in this case, offloading the computation does not ease the overall computational burden so as to make free computational resources available. Importantly, the efficiency gains occur predominantly in purely theoretical scenarios (as in the case of the quantum factoring algorithm), and are limited to cases when direct physical intervention on a dynamical system replaces conventional digital simulation of a physical system. Whether such simulation may play a crucial role for practical control purposes in robotics, for example, remains a very open question.

Even if the agent’s morphology could be used for computational purposes, it would be surprising to see that, say, muscles, compute significantly faster than neurons. After all, neurons, for all we know, were evolutionarily selected *for* computation, and muscles for executing movement. (It is also far from clear how reliable hybrid computational architectures (for example, ones including muscles and neurons) would be in comparison to pure neuronal architectures.) Of course, if my argument is correct, it would also be surprising to see that muscles compute in an overwhelmingly slower way, for example exponentially slower.

Arguably, morphology may indeed facilitate solving some tasks, as in direct physical preparation of a chemical system instead of digital computing. However, it does so mostly by obviating the need for computation (such as simulating chemical interactions digitally), rather than by offloading it. In such cases, it is exactly the case that less computation is needed; the physical structure can play a crucial role, and morphology could be a game changer. One could be tempted to metaphorically say that the initially planned computation was “offloaded,” but such talk is confusing (cf. Reference [7], pp. 2–3 for a similar criticism).

Let us return to the case of the robotic cricket. The ears of the cricket are mostly sensitive to a selected range of frequencies (only between 4 to 5 kHz), and are highly directional [45]. Their high directionality eases the task of phonotaxis, or tracking the source of the calling song of another cricket. Thus, replicating the ears of the cricket directly by building a physical replica is a computationally good idea. Instead of using a standard unidirectional microphone with a range between 20 Hz to 20 kHz, which would imply that a high number of input signals from the microphone have to be filtered out computationally, including background noise, one can rely on the cricket’s morphology. However, this is exactly what obviates the need for filtering. Literally, no computation is “offloaded”; rather, the filtering computation is no longer required at all, which obviously contributes to higher computational efficiency. Here, it must be noted, the early proponents of morphological computation, Pfeiffer and Bongard, were rightly cautious: “the concept of morphological computation still awaits qualification: how much computation is really done by a spring in the joint, or a change in morphology? Or perhaps this is not the right question.” [11] (p. 361-2). Indeed, it is not.

If my analysis is correct, then what is essential for explaining the higher efficiency in the cricket robot is both the morphology of perceptual organs and the *lack* of computation, but not morphological computation. Indeed, morphology facilitates the task because it is specified computationally in a simpler manner. At the same time, the hearing organ is part of the computational machinery of the cricket, so it has a computational role to play. However, it is not this role that is crucial. Rather, the crucial fact is that their bodily features allow crickets to avoid too much perceptual computation. Nonetheless, in this case, the notion of offloading may be used, at best, metaphorically. Again, it transpires that it is not morphological *computation* that is essential. 

Of course, the morphology of animals (or robots) is important in understanding or explaining their functioning. This is hardly surprising or theoretically novel, although it may be studied more clearly from the perspective of control theory (hence, I concur with Reference [7] that the term morphological *control* is much less confusing). What is difficult and theoretically significant is determining how the benefits of using morphology to interact with the environment can be precisely measured (for quantitative proposals, see References [3,59]).

The analysis in this section suggests that the impact of morphology on computational efficiency varies, compared with conventional computing. First, there are cases of hybrid systems, such as Paul’s XOR robot. These robots may use their morphology to perform tasks that otherwise would have to be performed using silicon chips, but classical complexity theory suggests it is highly unlikely that they would outperform silicon chips. Second, there could be direct physical models, as in the chemical interaction example studied in Reference [14]. At least sometimes, such physical models can be manipulated much more efficiently than their conventional digital simulation operates. However, it remains unclear whether and how frequently such efficiency can be practically used for control purposes. Third, the need for computation may be obviated, as in the case of the hearing organ of the cricket robot.

Explanatorily, therefore, the stress on morphological computation turns out to be blown out of proportion in recent debates, as the role of such computation in efficiency is not unified. Perhaps the term itself it is a misnomer, and what is at stake is the body morphology *simpliciter* and its informational exploitation in control or direct physical modeling. The terms *morphological control* or *direct physical modeling* seem, therefore, much better suited to such discussions.

In the following section, another purported role of morphological computation is analyzed: the role of accounting for biological adaptivity by analyzing the morphology of an agent as a direct model of the environment. That would be, after all, no small feat.

## 4. Models of Environment versus Computational Models of Environment

In this section, the focus will be on a recent proposal to treat agents as embodied models of their environment which they literally use to compute. Unfortunately, this proposal conflates all observer-available information with information available to systems under study. It will be argued that the exclusive focus on morphology is detrimental without analyzing the kind of control structures that are available in a given biological system.

The proposal analyzed in this section stems from the account of biological adaptivity in terms of the free-energy principle (FEP) [60]. While both the exact formulation of the theory and its justification remain a complex issue (for a recent review of the role of the FEP, see Reference [61]) in this analysis, only the possible connection of this account to morphological computation will be studied.

According to Karl Friston, the FEP brings to the fore the critical role of embodiment in cognition:

Not only does the agent embody the environment but the environment embodies the agent. This is true in the sense that the physical states of the agent (its internal milieu) are part of the environment. In other words, the statistical model entailed by each agent includes a model of itself as part of that environment.

([62], p. 89)

Friston takes the morphology of agents to statistically model the environment. This is probably due to the fact that the agents are adapted to the environment. The most surprising claim is, however, that “an agent does not have a model of its world—it *is* a model. In other words, the form, structure, and states of our embodied brains do not contain a model of the sensorium—they are that model.” ([63], p. 213). Most likely, Friston embraces the idea of morphology facilitating control in this passage. However, he seems to claim that because agents are the mirror of their environment, which is what the FEP is supposed to necessitate, these models also drive the agency of all living organisms. The same claim is also embraced in Reference [10]. Pezzulo et al. suggest that one could reinterpret the good regulator theorem [64] to say that “a good controller needs to *be* (not necessarily to *include*) a model of a system—hence, bodily and morphological processes can be part and parcel of the model.” ([10], p. 11).

In the same short commentary, Friston gives a simple example. In his view, two pathways of visual processing in the brain are predictable from the FEP:

if anatomical structure in the brain recapitulates causal structure in the environment, then one would expect independent causes to be encoded in functionally segregated neuronal structures. Given that objects can be in different places, they possess separable attributes of “what” and “where.” This translates into separate neuronal representations in segregated visual pathways.

([63], p. 213)

This small passage shows a number of serious flaws in the way Friston presents his account of the FEP. First of all, the FEP does not, to be exact, *predict* the structure of the sensory pathways, as we already know that there are two streams of visual processing. It is also difficult to say how to justify such *expectations* by referring to the FEP. Moreover, while it has also turned out to be true that two streams are also ascribed to auditory processing [65] (even though, as Reference [66] stresses, this is a huge simplification), as the FEP would predict, there is no reason to suppose that there are two pathways for, say, olfaction (even though there are two systems in olfaction, their functions differ significantly, cf. Reference [67]). Moreover, this does not seem to be the feature of all biological brains (only primates seem to manifest such functional separation). Thus, the FEP is presented in a way that suggests it generates false predictions (or gives rise to false expectations), at least in this little passage. Friston seems to jump from the hypothesis “anatomical structure in the brain recapitulates causal structure in the environment” to a particular claim about the mechanisms of the brain. The jump is all the more visible when we consider that the FEP does not even include the term “visual,” so it cannot, in reality, predict anything about vision, and it is actually difficult to say what he means by the verb he uses: *expect*. To infer or expect this functional separation, one has to assume much more than the FEP.

However, the problem runs even deeper: the initial hypothesis about the recapitulation cannot be true. There are brain waves, for example, and not everything that the brain represents and responds to are waves. Moreover, if brains are capable of executing probabilistic reasoning (variational Bayes, according to Friston), mechanisms of this reasoning need not recapitulate *anything* in the environment. In other words, agents, even if they model their environment—which is plausible otherwise, for example owing to the good regulator theorem [64]—they are not merely mirrors of their environment.

Moreover, even if one could expect some difference in the way two properties of visual objects are processed in the brain, because these are two different properties of objects as we see them, there is no reason to expect separate *pathways*. There could be just processing stages, as with color and edge detection. After all, colors are not what objects *are*, so why not hypothesize a larger number of dissociable pathways? More importantly, why not hypothesize that there should be a pathway that responds to magnetic properties of visual objects, and another one responsible for perceiving their radioactivity?

One obvious response—that there are no specialized sensors of radioactivity in humans—is not available to Friston. There are two reasons. First, visual location has no specialized sensor. It is the light that the human retina is sensitive to, and the information about object location has to be picked up from the light. Second, Friston understands sensory states of organisms very broadly; anything that lies at the boundary of organisms, defined more formally as one of their Markov blankets [68], is a sensory state. In this sense, any organism is sensitive to all kinds of physical energy surrounding it. Thus, if the brain of a human being should also recapitulate the structure of the environment, it should sport radiation pathways, ultraviolet light pathways, and electricity pathways. After all, our bodies do respond causally to these forms of energy, so this is undeniably part of the interaction of the human organism with its surroundings at its Markov blanket. Humans may suffer acute radiation syndrome, for example, so their bodies are, in some sense, mirrors of the radiation they were exposed to.

However, this kind of model, as embodied in the leukemia that one might suffer after radiation exposure, is not poised for informational use the same way as the light waves entering the retina are. As far as we know, there are no generative models of radiation in the brain, in contrast to generative models of visual objects we encounter. Thus, there is an equivocation hidden in the very claim that bodies are probabilistic models of their environment, because it suggests strongly that statistical inference of agents can rely on such models. In truth, however, only external observers (including the radiated human being equipped with appropriate measuring devices) can treat traces of radiation as models of the surrounding. They are observer-dependent models, not “animal-perspective” models. [69] 

The distinction between the observer perspective and the animal perspective, elucidated by Chris Eliasmith in Reference [69], is crucial in this context. Eliasmith stresses that instead of focusing on the conditional probability of the stimulus *s*, given a response *r*, *p(s|r)* has been wrongly ignored by analyzing the converse, *p(r|s)*. The distinction between two perspectives also tracks the distinction, or so I will argue, between information available for computational processing outside the animal, and information available for processing within it. While one can, for example, make natural information part of a computational process, e.g., by including a thermistor or any kind of sensor in a computational system, the indicator, such as a thermometer (or a thermistor), need not be processed by the animal itself. One can measure the temperature of a frog with a thermometer, and the frog will be perfectly oblivious to the thermometer’s reading (unfortunately, again, its anatomy somehow fails to recapitulate this part of its surroundings). The thermometer may interact with the frog, but not in the way the frog’s own sensors or receptors do.

The easiest way to elucidate the distinction further is to focus on biological organisms as regulators, or controllers, of their actions. It is quite clear that only some part of causal interactions of these organisms with the environment constitute their negative feedback as related to control; by seeing the organisms that can control (not just respond causally to), one can theorize about their proper modeling relationships. Thus, from this perspective, it is obvious that organisms that can differentially control the location of objects (by moving them) and plan their own actions depending on the category of an object should have access to these pieces of information separately. However, it does not follow that they should have differing processing pathways or stages, only separate informational states. Furthermore, because organisms, as far as we know, cannot control objects depending on their radioactivity, even if their radioactivity is traceable in their body morphology, it is not part of their own informational economy.

Thus, there is one special sense of morphology facilitating control when it gives rise to informational relationships. In the case of cricket ears, crickets do not respond differentially (do not control their own actions) to sounds that lie outside their hearing range. Their morphology actually inhibits certain informational relationships from occurring: it facilitates control indirectly, by limiting the amount of information poised for motor control in cricket phonotaxis. In contrast, the information conveyed by their ears facilitates perception. This is a clear sense of facilitating control via perception. Thus, this kind of morphological computation is genuine physical computation. Similarly, control information, as involved in metabolic control—evolutionarily important according to the current theories of nervous system evolution [70]—may involve information as embodied in bodily states, and constitute hybrid computation, especially in organisms devoid of specialized nervous systems. This, again, is a genuine kind of physical computation according to our analysis from Section 2.

So, even though Friston does not use the notion of morphological computation, he seems to conflate three senses distinguished in Reference [7]. The same conflation is found in Pezzulo et al. [10], who explicitly link morphological computation with the idea that agents are their own models. While a number of physical entities may bear information about the milieu in which they are found, it need not mean that this information is available for them to process. Mere presence of physical processes that lawfully covary with the processes in their surrounding is not sufficient evidence that they are engaged in morphological computation as computational models of the environment. 

Friston’s conflation shows clearly that the problem of focusing on morphological computation is not merely terminological or verbal. In contrast to Pezzulo et al., he does not use the term *morphological computation* at all. However, there is a temptation that many theorists fall prey to: treat all observer-available information as information available to organisms under study. The exclusive focus on morphology is particularly detrimental without analyzing the kind of control structures that are available in a given biological system. In contrast, in Reference [3], two separate measures of morphological computation are offered, and formal analyses of both include the information that is available to the system. In particular, morphological computation is studied there either as (i) a negative effect of the action, or as (ii) positive effect of the world. In contradistinction to Friston’s proposal, the sensory part of the sensori-motor loop is included in various measures of both types of morphological computation (and it is not conflated with a Markov blanket of an agent).

To summarize, in this section, it was shown that the kind of theorizing that underwrites the enthusiasm shared by some proponents of morphological computation is actually unhelpful in understanding the organisms or robots under study. Computational theories and explanations of biological control, including biological cognition, should focus on the information as available to the organisms themselves. Again, this shows that morphological computation is not a special embodied perspective, and that it may actually obscure our understanding of the role of embodiment, of functional roles of anatomy of organisms, if we focus exclusively on the morphology without analyzing the informational relationships closer.

## 5. Conclusions

In this paper, it was claimed that the notion of morphological computation is not helpful in analyzing the contribution of the physical to computation. Moreover, the notion does not contribute to the explanation of the role of embodiment in cognitive agents. As analyses performed by Müller and Hoffman [7,49] show, the notion is actually disunified, and it could raise serious doubts about whether it constitutes a natural kind. Simply, it does not seem to carve the physical nature of computation at its joints because morphology merely facilitating control (without facilitating perception) is not computational at all. For this reason, it offers a suspicious kind of theoretical unification, one that is based on equivocation rather than fact.

In addition, the analyses of offloading tasks to the morphology of an agent seem to lack the rigor required to show that it is morphological computation proper that explains the success of “offloading.” Again, the contributions are disunified; sometimes, morphological computation may be arguably slower (when performed by an XOR-robot, for example). Sometimes, it seems that the *lack* of computation is responsible for success, for example in insects simply unable to respond to a class of perceptual stimuli. Finally, it could be more efficient due to the use of a physical system as its own direct model.

Lastly, it was argued that the problem with morphological computation is not just terminological. Leading theorists of adaptivity, who claim that agents are embodied models of their surroundings, fail to observe exactly the same distinction as the one that creates the gap between morphology facilitating control and other kinds of morphological computation. In other words, morphological computation is a notion that offers a superficial and merely verbal kind of unified perspective on natural cognition. However, even when the notion is made more precise, its contribution fails to be novel or theoretically fecund. In other words, the notion may be confusing in the worst cases. At best, however, it is nothing but physical computation.

Still, the current criticism should not be understood as a wholesale rejection of the study of various physical trade-offs among energy efficiency, speed, reliability, durability, or noise levels of physical computation. Not at all. Physical computation remains a physical phenomenon and cannot be studied purely in the abstract way. Its physical implementation does matter. However, introducing new terminology to stress that only one kind of computation, morphological computation, requires analysis of the physical impact of hardware is simply misleading. In cognitive (neuro)science, for example, all kinds of cognitive processing require this kind of attention to physical detail [13]. 

Morphological computation is nothing over and above physical computation. The proper study of physical computation includes the study of various physical substrates and the way they operate, including quantum systems, reservoirs, or biological mechanisms. Note that extant formal accounts of morphological computation are based on general accounts of physical computation (cf. References [7,14,16]). Unfortunately, these accounts are faulty and cannot address the problem of trivialization (see Section 2). Even if the mechanistic account of computation was introduced informally (Defenders of mechanistic explanation rely on formally well-studied notions of interventionist causation [71], information theory, and computability. The notion of mechanism is itself understood in terms of a relational part-whole structure with causal interactions. Hence, formalizing the framework presents no unsurmountable problems. Yet such a task is beyond the scope of this paper.) here, it has the resources to decide systematically whether a given mechanism is computational, and suggests that the role of the physical substrate cannot be neglected.
